# Effects of Atmospheric Plasma Corona Discharge on *Agrobacterium tumefaciens* Survival

**DOI:** 10.3390/microorganisms10010032

**Published:** 2021-12-24

**Authors:** Yulia Lazra, Bharath Gandu, Irina Dubrovin Amar, Efrat Emanuel, Rivka Cahan

**Affiliations:** 1 Department of Chemical Engineering and Biotechnology, Ariel University, Ariel 40700, Israel; Yulia.Lazra@gmail.com (Y.L.); bharathgandu@gmail.com (B.G.); irinadu@ariel.ac.il (I.D.A.); efiem80@gmail.com (E.E.); 2 Department of Environmental Studies, University of Delhi, New Delhi 110007, India

**Keywords:** cold plasma, *Agrobacterium tumefaciens*, bacterial viability, membrane permeability, reactive oxygen species

## Abstract

Soil-borne pathogenic microorganisms are known to cause extensive crop losses. *Agrobacterium tumefaciens*, a member of the Proteobacteria, causes the neoplastic crown gall disease in plants. Plant protection is mainly based on toxic chemicals that are harmful to the environment. The use of cold atmospheric-pressure plasma is an attractive method for microbial eradication. Its antimicrobial mechanism includes the formation of large quantities of reactive oxygen species (ROS). The advantages of eradicating bacteria using cold plasma are not needed for chemicals, short treatment, and environmental temperatures. This study examined the impact of plasma corona discharge exposure on *A. tumefaciens* viability, membrane permeability, relative cell size, and ROS formation. The results showed that 90 s of plasma exposure led to a reduction by four orders of magnitude when the initial concentration was 1 × 10^7^ CFU/mL and in a dry environment. When the initial concentration was 1 × 10^6^ CFU/mL, 45 s of exposure resulted in total bacterial eradication. In a liquid environment, in an initial concentration of 2.02 × 10^6^ CFU/mL, there was no complete bacterial eradication even at the most prolonged examined exposure (90 s). The influence of plasma treatment on the membrane permeability of *A. tumefaciens*, and their possible recovery, were analyzed using flow cytometer analysis using propidium iodide (PI). When the plasma-treated bacteria were suspended in Luria–Bertani (LB) (rich medium), the PI-positive count of the plasma-treated bacteria after two hours was 12 ± 3.9%. At the 24th hour, this percentage was only 1.74 ± 0.6%, as the control (0.7 ± 0.1%). These results may indicate the repair of the plasma-treated bacteria that were suspended in LB. At the 24th hour, the relative cell size of the treated bacteria shifted to the right, to ~3 × 10^4^ forward side scatter (FSC), about 0.5-fold higher than the untreated cells. Measurement of the ROS showed that the intracellular fluorescence of the 90-s plasma-treated cells led to significant fluorescence formation of 32 relative fluorescence units (RFU)/cell (9 × 10^4^ fold, compared to the nontreated cells). This study showed that cold plasma is a useful method for *A. tumefaciens* eradication. The eradication mechanism involves ROS generation, membrane permeability, and changes in cell size.

## 1. Introduction

Soil-borne pathogenic microorganisms may cause extensive crop losses by restricting water and nutrient uptake by the roots or decreasing the crop’s quality [1], which ultimately affects global agricultural productivity [2]. The major bacterial phytopathogens include *Streptomyces scabies*, *Ralstonia solanacearum,* and *Agrobacterium tumefaciens* [3]. *A. tumefaciens* is a rod-shaped Gram-negative soil bacterium, a member of the Proteobacteria within the family Rhizobiaceae [4], closely related to symbiotic species of nitrogen-fixing rhizobia [5]. It is a facultative phytopathogen able to cause neoplastic crown gall disease on plants. Its phytopathogenic properties depend on the presence of a specific plasmid known as the Ti (tumor-inducing) plasmid [5]. Crown gall disease causes extensive damage in many agricultural species, such as those from the families Rosaceae (rose, apple, cherry, and pear), Vitaceae (grape), and the genus Juglans (walnut) [6].

Plant protection mainly includes chemical methods based on toxic chemicals that kill pathogenic bacteria, fungi, and pests living in the soil [7]. However, there are several disadvantages: the chemicals absorbed into the crops also pollute the soil and water sources [8,9]. The standard physical treatment method is based on covering the soil with plastic sheets in the hot season, preventing land use for about two months [10]. Physical methods for microbial eradication also include exposure to pulsed electric fields [11,12] and cold atmospheric-pressure plasma [13]. Plasma is the “fourth state of matter,” an ionized gas containing positively and negatively charged particles in an equal number. Cold plasmas are defined by the high temperature of electrons relative to the heavy particles. Low-temperature plasma discharges can be classified into atmospheric-pressure low-pressure plasma (the latter created under a low vacuum of 0.1–0.5 Torr) [14]. There are different types of equipment for generating plasma, such as the dielectric barrier discharge, atmospheric-pressure plasma jet, gliding arc, and corona discharge reactors [15].

Studies have shown that cold plasma could be applied to modify organic and synthetic surface properties [16,17,18] and for the treatment of agricultural seeds [19]. Cold atmospheric-pressure plasma is also an attractive method for microbial eradication since it operates at low temperatures [20]. Its eradication efficiency depends on gas composition, power input, and exposure mode [21,22,23,24,25]. The antimicrobial effect and mechanism of plasma disinfection include the formation of large quantities of reactive oxygen species (ROS), including atomic oxygen (O), hydroxyl radicals (·OH), hydrogen peroxide (H_2_O_2_), ozone (O_3_), and singlet oxygen (^1^O_2_) [26,27,28], as well as multiple reactive nitrogen species (RNS) such as ^•^NO (nitric oxide) and ONOO^−^ (peroxynitrite). These molecules play a significant role in the plasma biocidal process by altering the cell-wall components, the functions and structure of the phospholipid bilayer, the structure of nucleic acids and cellular proteins, gene expressions, and protein synthesis [26,29,30]. It was shown that cold plasma reduced the colony-forming unit (CFU) in *Staphylococcus aureus* biofilms within 6 min [31]. Inoculated Gram-positive *Bacillus subtilis* on black peppercorn surfaces were reduced by five orders of magnitude within 300 s, while the Gram-negative *Salmonella enteritidis* were totally eradicated by seven orders of magnitude [32].

Eradication of bacteria using plasma treatment has some advantages over other conventional methods, including there being no need to use chemicals, and shorter times needed for treatment [33]. In addition, the antimicrobial activity takes place at around room temperature, which allows for its utilization on thermal-sensitive surfaces [34].

This research investigated the effect of plasma corona discharges on the eradication of *A. tumefaciens* by impacting its viability, membrane permeability, relative cell size, and reactive oxygen formation.

## 2. Materials and Methods

### 2.1. Plasma Corona Discharge System and Experimental Conditions

**Figure 1:**
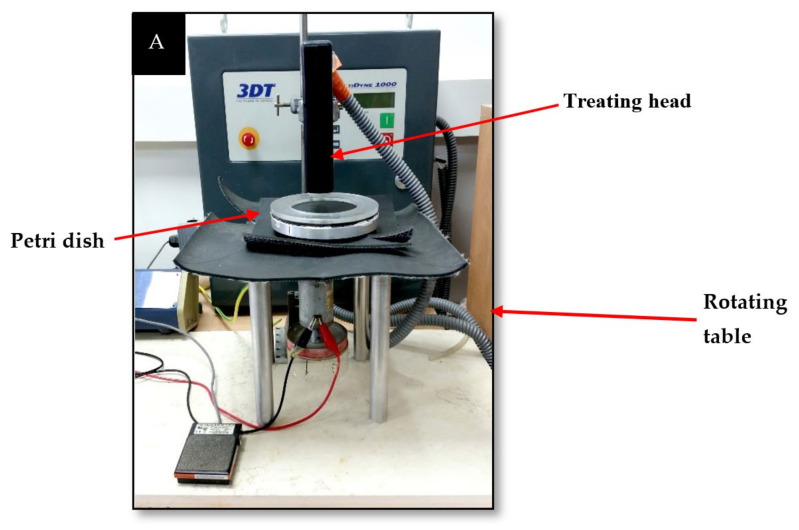
Plasma corona discharge device.

The plasma corona discharge device (3DT, Multidyne 1000, Germantown Wisconsin USA) consisted of two hook-shaped wire electrodes that served as a treating head. The physical conditions for generating plasma are high voltage at the electrode (2 × 12 kV and 50 Hz; output voltage: 24VDC maximum; and output current: 0.3 AMPS maximum) with ambient air as a carrier gas at atmospheric pressure (measurements of the voltage between the anode of the plasma device and the cathode of the sensor, and the UV intensity generated by the plasma corona device, are shown at Supplementary Materials Figures S1–S3). A rotating table (15 × 15 cm), made of steel coated by polyvinyl chloride (PVC), was adjusted under the treating head at a distance of two cm. The table was connected to a power supply (PowerPacTM Basic, Bio-Rad) to enable a rotation rate of 11 rpm. As described in our previous study, this is the optimal rotation rate for soil bacterial eradication [13]. The bacterial sample was spread in a Petri dish that was placed at the center of the rotating table (Figure 1). 

### 2.2. Growth Conditions

The *A. tumefaciens* was acquired from the Volcani Institute (Israel) and was grown in Luria broth (LB) agar for 24 h at 30 °C. Isolated colonies were suspended in 30 mL LB, incubated for 2 h at 30 °C, and agitated at 70 rpm to reach the log phase. The culture was centrifuged at 10,000× *g* for 10 min, and the sediment was washed with phosphate-buffered saline (PBS). The bacterial sediment was resuspended in PBS, to a final optical density of 0.01–0.3 at 590 nm, which was determined using a spectrophotometer (Genesys 10S UV-VIS, Thermo Scientific, Waltham, MA, USA).

### 2.3. Preparation of the Bacterial Sample for Exposure to Plasma Corona Discharge

The culture (0.01–0.3 at 590 nm) was divided into portions of 1 mL, inserted into Eppendorf tubes, and centrifuged at 10,000× *g* for 10 min. The sediment was washed with PBS, resuspended in 30 µL PBS, placed in the center of a Petri dish, and allowed to dry for 1 h at room temperature. The Petri dish with the dry bacterial layer was exposed to a plasma corona discharge for 0–90 s, as described above.

### 2.4. Viable Count Assay

The treated sample was collected in 1 mL PBS into an Eppendorf tube, vortexed, and serially diluted. The appropriate dilutions were spread on LB agar plates and incubated at 30 °C for 24–48 h. Viable cells were determined by counting the CFU and multiplying them by the corresponding dilutions. The same procedure was performed for the control sample, excluding exposure to plasma. The results of the CFU were calculated for 1 mL suspension.

### 2.5. Cellular ROS Detection

The levels of intracellular reactive oxygen species (ROS) were measured using the fluorescent dye 2′,7′- dichlorodihydrofluorescein-diacetate (H_2_DCFDA) assay kit (Abcam, Burlingame, CA, USA). The bacterial sample was prepared and exposed to plasma as described above. The treated and nontreated samples (about 3 × 10^8^ CFU/mL) were collected, and each was suspended with 1 mL of buffer containing 10 µM H_2_DCFDA, followed by incubation in the dark for 30 min at 37 °C and 150 rpm. Then, the sample was sonicated in ice for 1 min followed by 30 s of rest (this step was repeated four times). A sample of 100 µL was inserted into the well. The fluorescence was measured in a 96-well plate flow cytometer reader (infiniteM200, TECAN, Grödig, Austria) with an excitation wavelength of 485 nm and an emission wavelength of 535 nm. Results were obtained by calculating the mean fluorescence of 4 independent replicates. The mean fluorescence was normalized to the number of live cells in the different treatments. For negative control measurements, the bacteria were grown in the absence of H_2_DCFDA, which allowed us to measure the fluorescence background. Positive control was prepared by adding 50 µM of tert-butyl hydrogen peroxide (TBHP) in a buffer containing 10 µM H_2_DCFDA to a nontreated sample, followed by incubation in the dark at 37 °C and 200 rpm for 4 h before the sonication stage.

### 2.6. Examination of Bacterial Membrane Permeability and Cell Size by Flow Cytometry (FCM) Analysis

The bacterial sample was prepared and exposed to plasma as described above. The 30-s plasma-treated bacteria (4 × 10^6^ CFU/mL) was collected in 1 mL DI water and centrifugated. The sediments were divided into two groups. The first was suspended in BPS (total 2 mL), and the second was suspended in the rich LB medium (total 2 mL). The control samples were prepared as described for the treated cells, only omitting the exposure to plasma. Immediately after the exposure (time 0), the fluorescent propidium iodide (PI) dye at a final concentration of 1.5 µM was added to a sample of 500 µL, followed by incubation for 5 min at 37 °C. The remaining bacterial suspensions in PBS (1.5 mL) or LB (1.5 mL) were incubated at 30°C and agitated (70 rpm) for 24 h. At 2, 4, and 24 h, the PI was added to a sample (500 µL) and prepared as described for the sample of time zero. The bacterial membrane permeability and relative cell sizes in the samples (about 50,000 cells each) were examined using flow cytometry (Beckman Coulter, Atlanta, GA, USA). Data were analyzed using FlowJo software (Tree Star, San Carlos, CA, USA).

### 2.7. Statistics

The data are expressed as means ±STDEV (standard deviation) of 3–4 independent replicates. The paired *t*-test was used for the estimation of statistical significance.

## 3. Results

### 3.1. The Effect of Plasma Corona-Discharge Exposure Duration on the CFU of A. Tumefaciens

**Figure 2:**
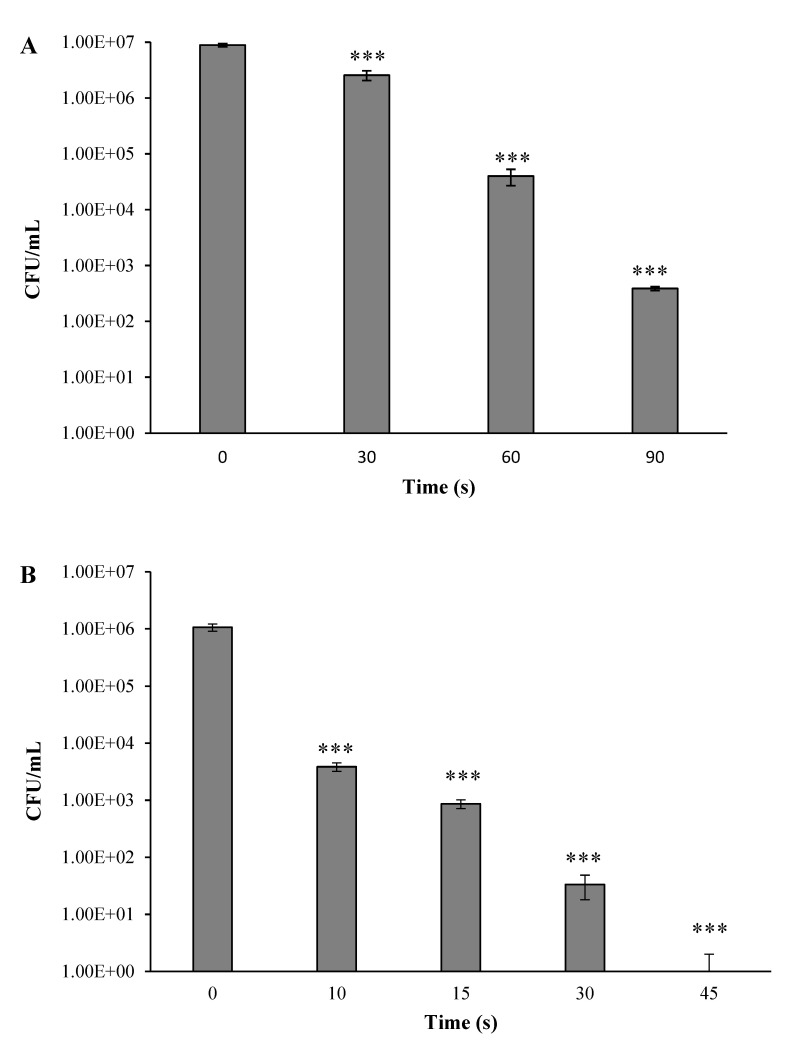

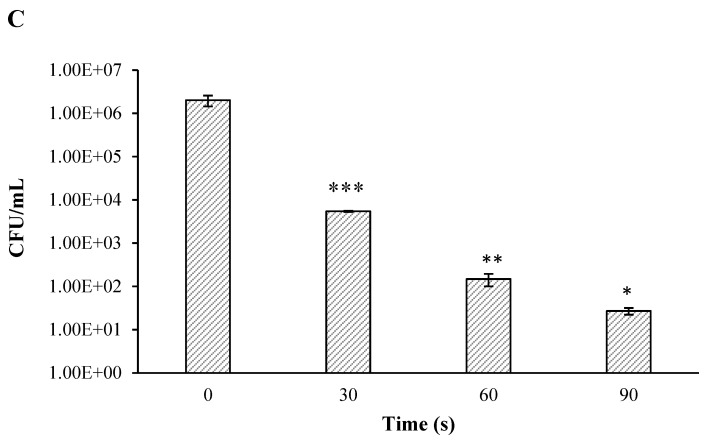
*A. tumefaciens* CFU/mL as a function of plasma corona-discharge exposure duration, in a dry environment with an initial bacterial concentration of 10^7^ CFU/mL and 10^6^ CFU/mL (**A**,**B**), respectively, and in liquid conditions with an initial bacterial condition of 10^6^ CFU/mL (**C**). Results are mean STDEV of four independent experiments. * *p* < 0.005, ** *p* < 0.005, and *** *p* < 0.0005, compared to control, determined by *t*- test.

The impact of the plasma corona-discharge exposure duration on the *A. tumefaciens* viability in a dry environment is shown in Figure 2A,B. The bacterial samples with two different initial concentrations of 10^7^ and 10^6^ CFU/mL were centrifuged, and the sediment was suspended in PBS. Each sample was spread in the center of a Petri dish, left to dry at room temperature, and then exposed to a plasma corona discharge for 10 to 90 s. A control sample was treated in the same manner, except for the exposure to plasma. At indicated times, the plasma-treated and nontreated samples were collated in PBS (1 mL). Each sample was serially diluted and pour-plated on LB agar. After 24–48 h of incubation, the CFU per ml was calculated for the experimental and control samples. The results showed that at an initial concentration of 10^7^ CFU/mL, plasma treatment of 30 s led to a minor bacterial reduction from 10^7^ CFU/mL (control) to 2.6 × 10^6^ CFU/mL (not a significant reduction). Treatment of 60 s led to a bacterial decrease by two orders of magnitude, to 4.0 × 10^4^ CFU/mL, and treatment of 90 s led to a bacterial reduction by four orders of magnitude, to 3.9 × 10^2^ CFU/mL.

When the initial concentration was 1 × 10^6^ CFU/mL (Figure 2B), a significant decrease in bacterial cells was observed. Plasma treatment of 10 s led to bacterial reduction of three orders of magnitude from 1.1 × 10^6^ (control) to 3.9 × 10^3^ CFU/mL. Treatment of 15 s led to a bacterial decrease by four orders of magnitude to 8.7 × 10^2^ CFU/mL. Treatment of 30 s led to a bacterial reduction by five orders of magnitude to 3.3 × 10^1^ CFU/mL, and treatment of 45 s led to total eradication.

The same experiment was performed except that the bacteria were suspended in liquid (2 mL PBS). It can be seen (Figure 2C) that increasing the plasma treatment duration led to a gradual decrease of the CFU/mL. Treatment of 30 s led to bacterial reduction by three orders of magnitude from 2.02 × 10^6^ CFU/mL (nontreated) to 5.44 × 10^3^ CFU/mL. Treatment of 60 s led to bacterial reduction by four orders of magnitude to 1.48 × 10^2^ CFU/mL. However, even after 90 s of plasma treatment, there was no total eradication of the bacteria: the CFU/mL was 2.7 × 10^1^. 

In summary, in the dry environment containing the initial concentration of 1 × 10^7^ CFU/mL, after 90 s of treatment there was a reduction by four orders of magnitude. When the initial concentration was 1 × 10^6^ CFU/mL, after 45 s of treatment, there was total bacterial eradication. In the liquid environment containing an initial concentration of 2.02 × 10^6^ CFU/mL, a treatment of 30 s reduced the bacteria reduction was by only three orders of magnitude. There was no total bacterial eradication even at the most prolonged examined treatment (90 s). It is important to note that exposure bacteria to cold low-pressure nitrogen plasma (90 s) did not lead to eradication (data not shown).

Guo et al. showed that cold plasma reduced the CFU in methicillin-resistant *Staphylococcus aureus* biofilms by 3.5 log10 within 6 min. It was found that the plasma treatment led to the polymerization of SaFtsZ and SaClpP proteins in the *S. aureus* [31]. Mošovská et al. exposed bacteria inoculated on the surface of black peppercorns to cold atmospheric plasma in ambient air for 300 s. This treatment reduced *B. subtilis* from 7.36 to 2.30 log10 CFU/g and reduced the *B. subtilis* endospores from 4.42 to 2.39 log10 CFU/g, while the Gram-negative *Salmonella enteritidis* and *Escherichia coli* were reduced below the detection level (1.0 log 10 CFU/g) from initial populations of 7.60 log10 CFU/g and 7.45 log10 CFU/g, respectively [32]. Cold atmospheric pressure plasma operating in argon was used to expose *Staphylococcus aureus* and *Listeria monocytogenes*, placed on gel discs. The results showed only a mild biocidal effect, with a maximum 1–2 log reduction after 120 s [35]. Ermis et al. examined a hybrid treatment using vaporized ethyl pyruvate and atmospheric-pressure plasma to inactivate *Bacillus cereus* and *E. coli* on fresh lettuce leaves. The combination of ethyl pyruvate (10 μL dm^−3^) with atmospheric-pressure plasma was more effective on the inactivation of bacteria, compared to each of the treatments separately; the total viable counts dropped by nearly five CFU/cm^2^ compared to the control [36]. Zhang et al. observed that a higher initial bacterial concentration was more resistant to inactivation by cold plasma. This phenomenon was attributed to the enhanced resistance by quorum sensing [37]. Mai-Prochnow et al. investigated the efficacy of cold atmospheric-pressure plasma on different bacterial species according to their cell wall thickness. Less than one log10 reduction of Gram-positive *Bacillus subtilis* biofilm occurred after 10 min of plasma treatment. This bacterium possesses a cell wall with a thickness of 55.4 nm. However, biofilms of Gram-negative *Pseudomonas aeruginosa* (cell wall thickness of 2.4 nm) were almost completely eradicated. Planktonic cultures of Gram-positive *Staphylococcus epidermidis* were more resistant to plasma treatment than the Gram-negative *Pseudomonas libanensis* [38].

In conclusion, bacterial eradication using plasma treatment directly depends on the bacterial type and initial concentration, the length of treatment, and the environmental conditions (dry or liquid).

### 3.2. A. Tumefaciens Viability and Possible Recovery of Corona-Treated Bacteria

The influence of corona plasma treatment on *A. tumefaciens* (10^6^ CFU/mL) and their possible recovery was examined by a viable count assay. The bacteria were exposed to plasma in a dry environment for 30 s. Immediately after the treatment, the bacteria were suspended in two different solutions: a rich LB medium (1 mL), and PBS (1 mL) where bacteria can only survive. This step was repeated five times, producing a total volume of 5 mL containing suspended bacteria in either LB or PBS. Each treated bacterial suspension was incubated for 24 h at 30 °C. At times of 0 (immediately after the treatment), 2, 4, and 24 h, a portion (100 μL) of suspended bacteria was serially diluted and pour-plated on LB agar, as described in the Methods section.

**Figure 3:**
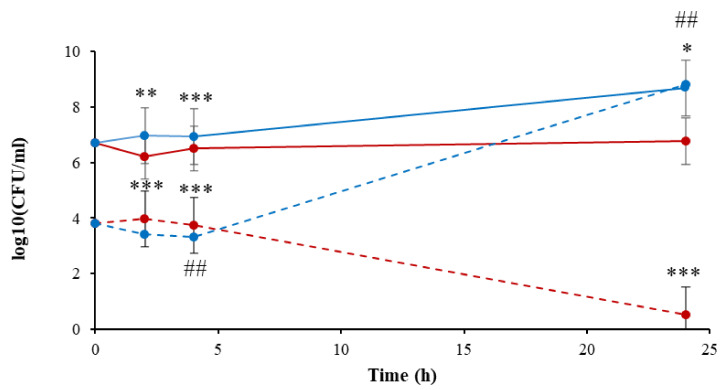
*A. tumefaciens* viability during 24 h, as a response to plasma treatment of 30 sec followed by suspension in PBS (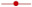) and LB (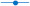), compared to the controls of the nontreated bacteria in PBS (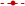) and LB (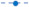). Results are the mean of SD of three independent experiments. *P*-value significance is presented by *t*-test of CFU/ml of the plasma-treated *A. tumefaciens* in LB, compared to the treated sample in PBS on the same selected time ## *p* < 0.01. *p*-value of the CFU/mL count in each examined time related to its control * *p* < 0.05, ** *p* < 0.01, and *** *p* < 0.001.

As shown in Figure 3, the initial concentration of the nontreated *A. tumefaciens* in PBS and LB was 5.1 × 10^6^ CFU/mL. In the nontreated bacteria suspended in PBS, this concentration remained about the same during the entire experiment. At the end of the experiment (24th hour), the bacterial concentration was 6.1 × 10^6^ CFU/mL. However, the suspended bacteria in LB continued to replicate, and after 24 h reached 4.95 × 10^8^ CFU/mL, two orders of magnitude higher than the control bacteria in PBS. Interesting results were observed for the plasma-treated bacteria. At time zero (immediately after the exposure to plasma), the initial concentration of *A. tumefaciens* in PBS and LB was 6.37 × 10^3^ CFU/mL, about three orders of magnitude less than the controls. The plasma-treated bacteria that were suspended in PBS stayed at about the same concentration for 4 h, and at the 24th hour after the treatment, total eradication was observed, while the plasma-treated bacteria that were suspended in LB continued to replicate and at the end of the experiment (24 h) reached 6.6 × 10^8^ CFU/mL, about the same concentration as the nontreated bacteria in LB.

These findings show that exposing *A. tumefaciens* to plasma led to their eradication by three orders of magnitude immediately after the treatment. When the bacteria were immediately suspended in PBS, about 5.6 × 10^3^ CFU/mL survived during the first four hours. We assume that these bacteria were also injured since total bacterial eradication was observed on the 24th hour. When the bacteria were exposed to plasma and immediately suspended in LB, the injured bacteria continued to multiply. We assume that the rich nutrients in the LB enabled a repair mechanism.

### 3.3. A. Tumefaciens Membrane Permeability as a Function of Plasma Treatment and Possible Recovery

**Figure 4:**
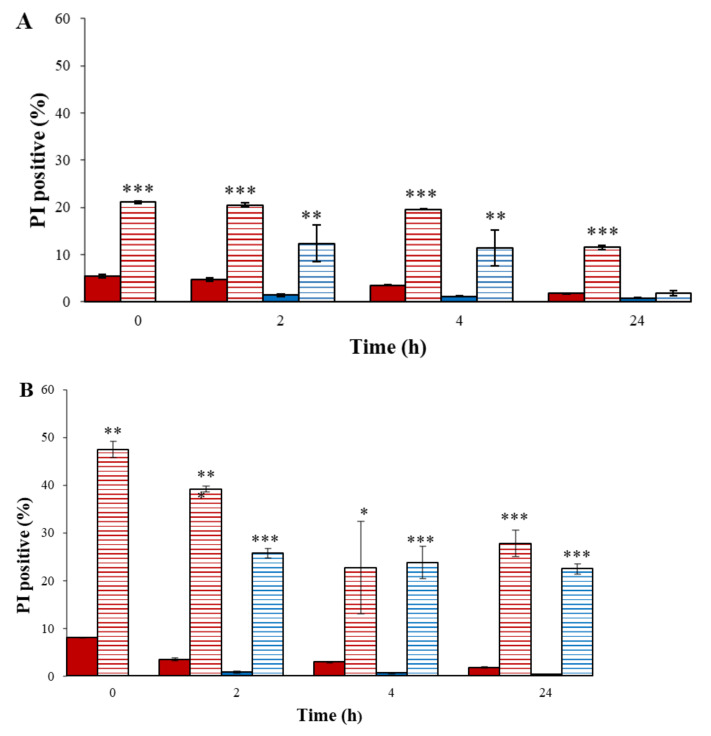
FCM analysis of *A. tumefaciens* membrane permeability as a function of plasma corona discharge exposure for 30 s. (**A**) and 45 s. (**B**). Nontreated *A. tumefaciens* in PBS (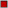) (0–24 h); plasma-treated *A. tumefaciens* in PBS (0–24 h) (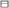); nontreated *A. tumefaciens* in LB (2–24 h) (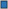); and plasma-treated *A. tumefaciens* in LB (2–24 h) (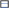). Results are mean SD of four independent experiments; *p*-value significance is presented by *t*-test of treatment compared to control: * *p* < 0.05, ** *p* < 0.001, *** *p* < 0.0001.

The influence of plasma treatment on the membrane permeability of *A. tumefaciens* and possible recovery was examined using FCM analysis. Samples of *A. tumefaciens* (4 × 10^6^ CFU/mL) were exposed to plasma (as described in Material and Methods) for 30 s and 45 s. The samples were collected and suspended in PBS or LB (1 mL each). This procedure was repeated five times, producing 5 mL for each of the suspensions. The suspensions were incubated at 30 °C and agitated at 70 rpm for 24 h. At indicated times, 0 (immediately after the exposure), 2, 4, and 24 h, fluorescent propidium iodide (PI) was added to the suspended bacteria. The samples were incubated for 5 min, followed by an examination of the membrane permeability using flow cytometer analysis (about 50,000 cells). The same procedure was performed for the control samples, except for omitting the plasma treatment (nontreated bacteria). The percentage of the PI-positive cells is shown in Figure 4A,B.

As shown in Figure 4A, the PI-positive cells of the nontreated *A. tumefaciens* in PBS at time zero were 5.4 ± 0.4%. This percentage stayed about the same for 24 h.

When the bacteria were treated for 30 s, the PI-positive cells of the plasma-treated bacteria in PBS at time zero were 21 ± 0.3%. This percentage remained steady for four hours, while at the 24th hour, it dropped to 11.5 ± 0.4%. We assume that at 24 h, many bacteria were lysed (as can be seen in Figure 3), and most of the sample included cell debris. It is important to note that the flow cytometer counts only cells (intact and injured) and not the debris of lysed bacteria. Thus, the results of the PI-positive cells (11.5 ± 0.4%) on the 24th hour were relatively low compared to expectations.

The PI-positive cells of the nontreated bacteria in LB after 2 h of incubation were 1.3 ± 0.2%, a percentage that remained about the same until the end of the experiment (24 h). When the plasma-treated bacteria were suspended in LB, the PI-positive cells of the plasma-treated bacteria after two hours were 12 ± 3.9%. At the 24th hour, this percentage decreased to 1.74 ± 0.6%, similar to the control (0.7 ± 0.1%). These results may indicate repair of the plasma-treated bacteria suspended in the rich LB medium.

The PI-positive cell count when the plasma treatment lasted 45 s is depicted in Figure 4B. The PI-positive cells of the nontreated *A. tumefaciens* in PBS at time zero were 8 ± 0.4%. This percentage stayed about the same for 24 h. Similar results were obtained for the nontreated bacteria that were suspended in LB. Different results were obtained when the cells were exposed to plasma for 45 s. At time zero, the PI-positive cells were about 50%. Over time, this count was progressively reduced, and by 24 h it was 30%, similar to the PI-positive cells that were suspended in LB. We assume that plasma treatment of 45 s was fatal to the cells, with no possibility for repair.

### 3.4. The Relative Cell Size of A. Tumefaciens as a Function of Plasma Corona Discharge Treatment

To examine the relative cell size, *A. tumefaciens* were exposed to plasma treatment for 45 s and then stained with PI. The samples were prepared as described for the examination for membrane permeability.

**Figure 5:**
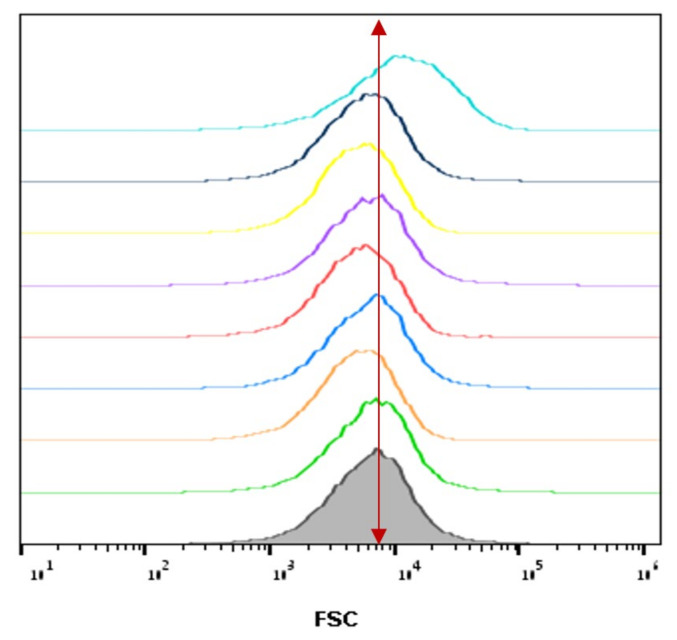
Relative bacterial cell size analysis of *A. tumefaciens*, plasma-treated for 45 s, and nontreated, at selected times (0, 2, 4, and 24 h.). Unstained cells: gray. Nontreated and plasma-treated bacteria at time zero: green and orange, respectively. Nontreated and plasma-treated bacteria at 2 h: azure and red, respectively. Nontreated and plasma-treated bacteria at 4 h: purple and yellow, respectively. Nontreated and plasma-treated bacteria at 24 h: turquoise and light blue, respectively.

As shown in Figure 5, the relative cell size of the non-treated *A. tumefaciens* in LB at time 0 was about ~8 × 10^3^ forward side scatter (FSC) (green), as well as at all the four selected times (0, 2, 4, and 24 h). The same relative cell size was observed for the plasma-treated cells at 0, 2, and 4 h. However, at the 24th hour, the plasma-treated cell size shifted to ~3 × 10^4^ FSC, about 0.5-fold higher than nontreated cells. These results indicate an increase in cell size during the 24th hour.

### 3.5. ROS Generation in A. Tumefaciens as a Function of Plasma Treatment

**Figure 6:**
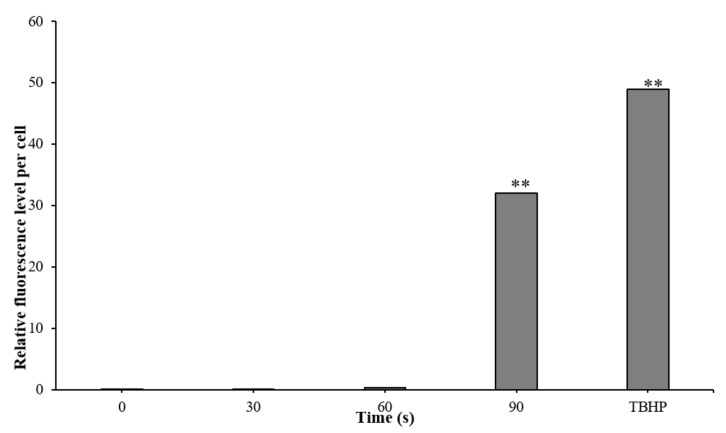
ROS level per *A. tumefaciens* bacterial cell as a function of plasma corona discharge treatment duration. Bacteria suspension (3 × 10^8^ CFU/mL) was treated with plasma for 0, 30, 60, and 90 s. The relative fluorescence level was normalized to the amount of the CFU in the different samples. TBHP served as a positive control. *p*-value by *t*-test: ** *p* < 0.005 presented in three independent experiments of exposure to 30, 60, and 90 s, compared to the non-treated cells.

ROS generation occurs as a by-product in living cells, as part of the standard metabolism through various pathways; it is also an environmental response to diverse stress triggers [27]. For measuring the ROS, the initial bacterial concentration was 3 × 10^8^ CFU/mL, as reported for the optimal concentration of this measurement [39]. *A. tumefaciens* were treated with plasma for 30, 60, and 90 s, except for the control (0 s). Each sample was collected and incubated with the fluorescent dye (H_2_DCFDA), followed by sonication as described in the Methods section. For the positive control, the samples were incubated with the addition of TBHP (tert-butyl hydrogen peroxide; Abcam, USA), a reagent known to induce ROS generation. The mean fluorescence level was normalized to the CFU in each sample, and the results are shown in Figure 6. The intracellular fluorescence level of the TBHP (positive control) per cell was 49 relative fluorescence units (RFU). The nontreated *A. tumefaciens* at time zero and 30 s of treatment showed about 0.001 RFU/cell. A treatment of 60 s led to 0.08 RFU/cell, and 90 s led to a significant fluorescence formation of 32 RFU/cell (9 × 10^4^ fold compared to the nontreated cells).

It was previously reported that cold plasma treatment induced the formation of different ROS species [26,27,28], which can cause cell leakage, lipid peroxidation, protein denaturation, DNA damage, and interference with cell metabolic activity [27,40]. The polyunsaturated fatty acids’ lipid membranes take part in the lipid peroxidation chain reaction under the action of ·OH and then transfer into lipid hydroperoxide [41], which causes cell membrane rupture and exudation of the intracellular material [42]. Yusupov et al. used reactive molecular-dynamics simulations to show interactions of plasma species with peptidoglycan. It was shown that plasma species can break structurally important bonds of peptidoglycan, ultimately leading to cell death [43].

However, in the presence of ROS, bacteria can develop protection mechanisms such as endospore formation, activation of stress-resistant genes, and production of adaptive mutations [44]. It was also reported that bacteria respond to ROS by reducing their surface area to decrease further damage, metabolic activities, and energy requirements [45,46]. Ahmed and Helmann showed that in *Bacillus subtilis*, hydrogen peroxide (H_2_O_2_) induced the expression of the PerR regulon, including catalase (KatA), alkyl hydroperoxide reductase, and the DNA-binding protein MrgA. They also had identified the P-type metal-transporting ATPase ZosA (formerly YkvW) as an additional member of the perR regulon. These regulons play an essential role in protection against oxidative stress [46]. Eiamphungporn et al. showed adaptive responses used by *A. tumefaciens* to overcome stress caused by peroxide. Exposure to low levels of peroxide and superoxide anions induced high levels of resistance to peroxide by this bacterium. It was found that superoxide (soxR) and peroxide (oxyR) regulators and a catalase gene (katA) are involved in these responses [47]. Storz and Imlay showed that the key regulators of the adaptive response in *Escherichia coli* are the oxidant-sensing transcriptional regulators OxyR and SoxRS, which regulate the inducible expression of antioxidant genes in response to H_2_O_2_ and superoxide anion exposure, respectively [48].

In summary, it was reported that ROS are involved in the mechanism of bacterial eradication using plasma. Our results also showed the generation of ROS in *A. tumefaciens* when exposed to plasma corona. However, when the plasma-treated *A. tumefaciens* were suspended in a rich medium, the bacteria continued to multiply, and by the 24th hour they reached to the same CFU/mL as the nontreated bacteria. We assume that the repair mechanism in *A. tumefaciens* is based on activating stress-resistant genes, as described by other studies.

## Supplementary Materials

The following supporting information can be downloaded at: https://www.mdpi.com/article/10.3390/microorganisms10010032/s1, Figure S1. Voltage decreases measurement system: scanner (A), power supply (B), UV filter placed on the sensor below the corona plasma source (C). Figure S2. UV light (%) transmission as a function of wavelength. Figure S3. UV intensity as a function of the distance between the cathode sensor and the electrode of the UV lamp and plasma corona device. UV lamp—dashed line, plasma corona device—solid line.
