# Supplementary material for: Effects of Atmospheric Plasma Corona Discharge on Agrobacterium tumefaciens Survival
Source: Microorganisms. 2021 Dec 24;10(1):32. doi: 10.3390/microorganisms10010032 (PMC8780683; doi:10.3390/microorganisms10010032)
Supplement: Supplementary file 1 [file microorganisms-10-00032-s001.zip › microorganisms-1475197-supplementary.pdf]

microorganisms

MDPI

Supplementary Material

Measurement of voltage between the anode of the plasma device and the cathode of the sensor that was connected to the system

The system consisted of a scanner device (Keythley 199 System, USA) (S1 A) connected to a programmable DC power supply (IDRC DSP-030-025HD, Taiwan) (Figure S1 B) and a Sb-Cs sensor (Hamamatsu R727, Japan) (S1 C). The power supply was set at a voltage of 15 V and a current of 1 A. In order to calibrate the system, units of resistance (Ω) were defined, and a series of experiments were performed in order to produce a difference of voltage between the anode of the device and the cathode of the sensor, without activating a corona plasma emission during its operation (ΔU) in the experimental range of 0.1 V- 1 V. Each of the experiments was performed with a change in the resistance values. The final values obtained were R1 = 500 kΩ and R2 = 80 kΩ. After the system was calibrated, the voltage (V) units were defined. The cathode of the sensor was placed under the treating head of the plasma corona device at different distances when a UV filter was placed on the cathode. The voltage decrease was measured both without the operation of the corona plasma device and during its operation.

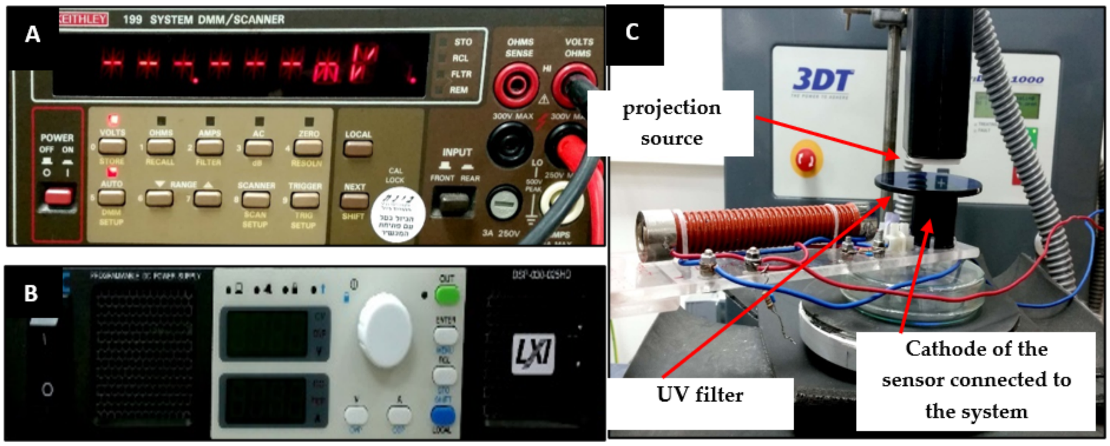

Figure S1. Voltage decreases measurement system: scanner (A), power supply (B), UV filter placed on the sensor below the corona plasma source (C).

Monitoring UV intensity generated by the plasma corona device

UV radiation is part of the decontamination activity of plasma corona. In the following experiments, the intensity of the UV light emitted from the plasma device was compared to the UV intensity emitted from a UV lamp, which is commonly used for bacteria disinfection. The examination was performed while measuring the UV lamp radiation intensity. The measured range in this lamp was between 180-650 nm, which necessitated using a filter to exclusively obtain UV radiation (the range of 200–380 nm).

To examine UV intensity generated from the plasma device and UV lamp, the range of the emitted light through the filter was analysed with a UV-Vis spectrophotometer (Varian Cary 100 Bio, USA) which was operated with and without the filter (Figure S2).

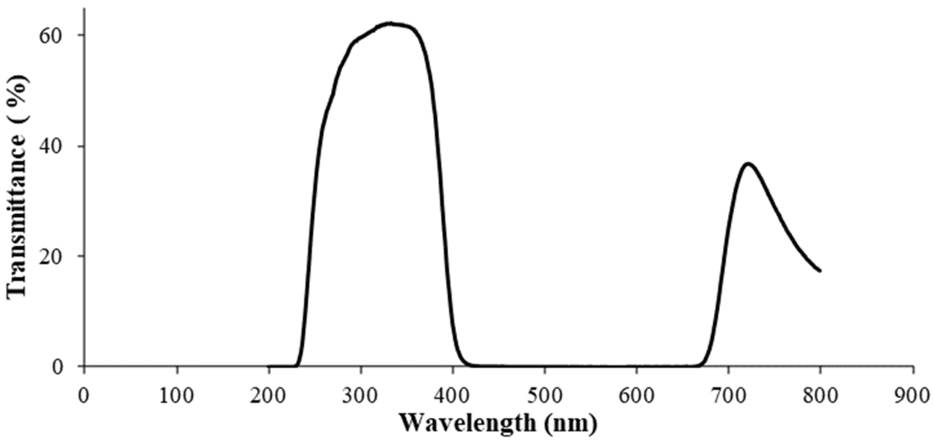

Figure S2. UV light (%) transmission as a function of wavelength.

As shown in Figure S1, the wavelength of the main light emitted through the filter was at 200-400 nm (UV light) and also slightly at 670-800 nm (infrared light).

To compare the intensity of the UV radiation emitted from the plasma corona device with that emitted by the UV lamp in the bactericidal range (200-400 nm), changes in the cathode voltage of the sensor were examined. The UV light filter was placed on the cathode, which was placed under the treating head of the plasma corona device and UV lamp. The measurements were performed without operating the plasma device or UV lamp, and again during their operation, at distances varying between 2 and 6 cm from the cathode sensor and the treating head. After the measurements, the UV intensity at each of the distances was calculated (Figure S3).

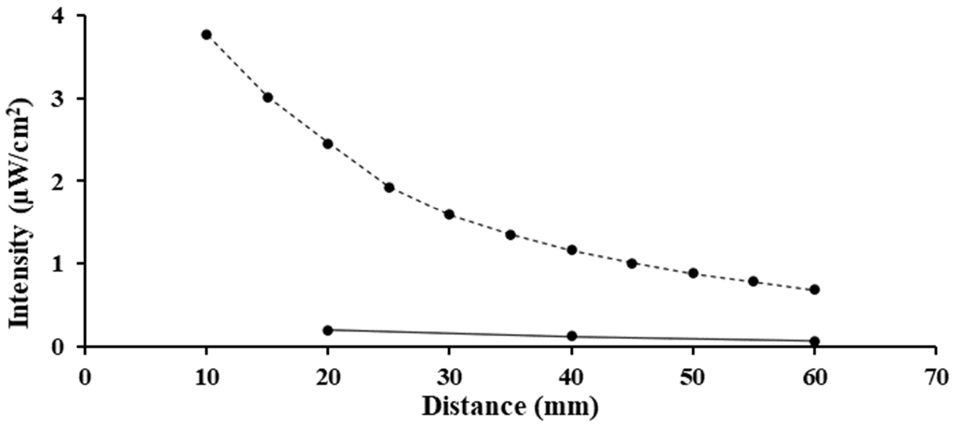

Figure S3. UV intensity as a function of the distance between the cathode sensor and the electrode of the UV lamp and plasma corona device. UV lamp - dashed line, plasma corona device - solid line.

As shown in Figure S3, the intensity of the UV radiation emitted from the UV lamp was significantly higher than that emitted from the plasma corona device. At a distance of 2 cm, the intensity of the UV radiation emitted from the plasma corona device was 0.21 μW/cm^2; at 4 cm, the intensity decreased to 0.13 μW/cm^2; and at a distance of 6 cm, the intensity was only 0.068 μW/cm^2. In contrast, UV radiation emitted from the UV lamp at a distance of 2 cm was 2.46 μW/cm^2, at 4 cm it was 1.16 μW/cm^2, and 6 cm the intensity was only 0.69 μW/cm^2. UV intensity decreased with increasing distance from the cathode of the sensor and treating head to the UV lamp or plasma corona device.

As mentioned, part of the plasma activity leads to the creation of charged particles, radicals, electromagnetic radiation, and UV radiation; all these together cause the decontamination effect of the plasma. It was found that the UV intensity level emitted by the UV lamp was 2.46 μW/cm^2, 12 times higher than that emitted by the plasma corona device (0.20 μW/cm^2). Therefore, UV radiation emitted during plasma corona activity is not the only factor influencing the bactericidal phenomenon effect; it is part of a synergistic activity in the entire system, including charged particles and radicals.
